# Perceiving Positive Facial Expression Can Relieve Depressive Moods: The Effect of Emotional Contagion on Mood in People With Subthreshold Depression

**DOI:** 10.3389/fpsyg.2021.535980

**Published:** 2021-11-11

**Authors:** Yuko Yamashita, Tetsuya Yamamoto

**Affiliations:** ^1^Mutsumi Hospital, Tokushima, Japan; ^2^Graduate School of Technology, Industrial and Social Sciences, Tokushima University, Tokushima, Japan

**Keywords:** emotional contagion, depression, subthreshold depression, depressive mood, facial expression, emotion, barriers to psychotherapy, access to care

## Abstract

Emotional contagion is a phenomenon by which an individual’s emotions directly trigger similar emotions in others. We explored the possibility that perceiving others’ emotional facial expressions affect mood in people with subthreshold depression (sD). Around 49 participants were divided into the following four groups: participants with no depression (ND) presented with happy faces; ND participants presented with sad faces; sD participants presented with happy faces; and sD participants presented with sad faces. Participants were asked to answer an inventory about their emotional states before and after viewing the emotional faces to investigate the influence of emotional contagion on their mood. Regardless of depressive tendency, the groups presented with happy faces exhibited a slight increase in the happy mood score and a decrease in the sad mood score. The groups presented with sad faces exhibited an increased sad mood score and a decreased happy mood score. These results demonstrate that emotional contagion affects the mood in people with sD, as well as in individuals with ND. These results indicate that emotional contagion could relieve depressive moods in people with sD. It demonstrates the importance of the emotional facial expressions of those around people with sD such as family and friends from the viewpoint of emotional contagion.

## Introduction

In recent years, it has been widely acknowledged that there are many people who are not diagnosed with depression, even though they have significant depressive symptoms (Bertha and Balázs, 2013). The presence of depressive symptoms is associated with psychological distress, even in nonclinical groups, and is a risk factor for the development of major depressive disorder (Cuijpers and Smit, 2004; Cuijpers et al., 2007). Moreover, depressive symptoms are related to functional impairment and an increased risk of suicidal thoughts (Balázs et al., 2013). Therefore, it is important to reduce depressive symptoms in people with subthreshold depression (sD) to prevent a worsening of those symptoms.

However, people with mental health problems such as sD display an unwillingness to seek professional help, and low rates of undergoing medical examination and counseling (Naganuma et al., 2006; Yoshikawa et al., 2017). Thus, it is possible that there are many people with sD who feel psychological distress in daily life, yet do not receive professional treatment or support.

Emotional contagion is one potential method by which to improve depression in these people. Emotional contagion is the phenomenon by which an individual’s affective state matches the emotional display of another person (Lundqvist and Dimberg, 1995; Hess and Blairy, 2001). This phenomenon is thought to occur through the process of mimicry and feedback (e.g., Hatfield et al., 2014). Previous studies have shown the occurrence of emotional contagion by the perception of others’ emotional facial expressions. Hess and Blairy (2001) reported that participants who perceived happy and sad faces showed a subjective emotional state that was consistent with the emotional facial expressions they perceived. Moreover, Lundqvist and Dimberg (1995) reported that emotional contagion also occurred for disgust and fear facial expressions, in addition to happy and sad faces. Furthermore, Olszanowski et al. (2019) empirically demonstrated the occurrence of emotional contagion in happy, angry, and sad faces, and interpreted their findings from the perspective of a mediating role of facial mimicry. Together, these studies show that perceiving another person’s facial expression can affect our own emotional state.

Given that perceiving a positive facial expression creates a positive emotion in the perceiver, emotional contagion could be applied to relieve depressive moods in people with sD. Depressive mood is a core depressive symptom and reducing this mood could therefore decrease individuals’ psychological distress and prevent the development of depression. Actually, Santos et al. (2013) have reported that the positive emotion is related to the improvement of the levels of depression. However, the effect of emotional contagion in people with sD has not yet been investigated, and its usefulness is not yet clear. Previous studies showed that people with major depressive disorder have impairment in recognition of emotional facial expression and facial mimicry (Wexler et al., 1994; Demenescu et al., 2010), so it is unlikely to cause emotional contagion and to relieve depressive mood by the perception of positive facial expressions. However, people with subthreshold depression who are not as severely depressed as them may show the moderating effect of emotional contagion on depressed mood.

Therefore, in this study, we investigated the effects of emotional contagion on mood in people with sD. To this aim, we used facial stimuli with happy and sad expressions, and examined whether the perception of stimulus type influenced emotional state. We selected these two types of facial stimuli because perceiving happy faces may be related to relieving the participants’ depressive mood, while, in contrast, perceiving sad faces may be related to the worsening of their depressive mood. In previous studies, each participant was presented with multiple emotional facial expressions and asked to rate their emotional states after the presentation of each facial expression (e.g., Hess and Blairy, 2001). However, when using this method, the emotional state caused by presenting an emotional facial expression may affect the emotional evaluation of the different types of emotional faces presented afterward. Therefore, in this study, we examined emotional contagion by presenting each participant with only one type of emotional facial expression and asking them to rate their emotional states before and after the presentation. This method could yield more sophisticated results regarding the effect of perceived facial expressions on emotional state. In addition, because emotional contagion is thought to occur through mimicry (e.g., Hatfield et al., 2014), we also measured the response of participants’ facial mimicry during the presentation of facial stimuli using facial electromyography (facial EMG) to support the occurrence of emotional contagion. We recruited undergraduate students because, in Japan, they often have mild to moderate depressive symptoms (Shiraishi, 2005). Our results provide useful insights about the attitudes of individuals who come into contact with people with sD, such as family, and friends.

## Materials and Methods

### Participants

Participants were recruited through brochures and in-class announcements. Fifty-four participants showed their intention to participate in our study and came to the laboratory. One participant who exhibited severe depressive tendency was interviewed to assess for a current health condition using the Japanese version of the Mini-International Neuropsychiatric Interview (MINI; Sheehan et al., 1998; Otsubo et al., 2005). This participant met the diagnostic criteria for major depressive episodes, and was therefore asked to discontinue participation in the study to ensure the participant’s safety. Moreover, one participant who was an international student was excluded from the analyses because our sample consisted of Japanese undergraduate students only. Three participants were excluded from the analyses because confounding factors might have affected their moods. For instance, they reported that “I laughed because the person of facial stimuli resembled my acquaintance,” “I was feeling up before the presentation of facial stimuli because I was looking forward to participate in this experiment,” and “I’m not good at looking at faces”. Finally, 49 undergraduate students from Tokushima University were included in the analyses (six males, 43 females; mean age=19.90years, *SD*=1.08years).

**Figure 1:**
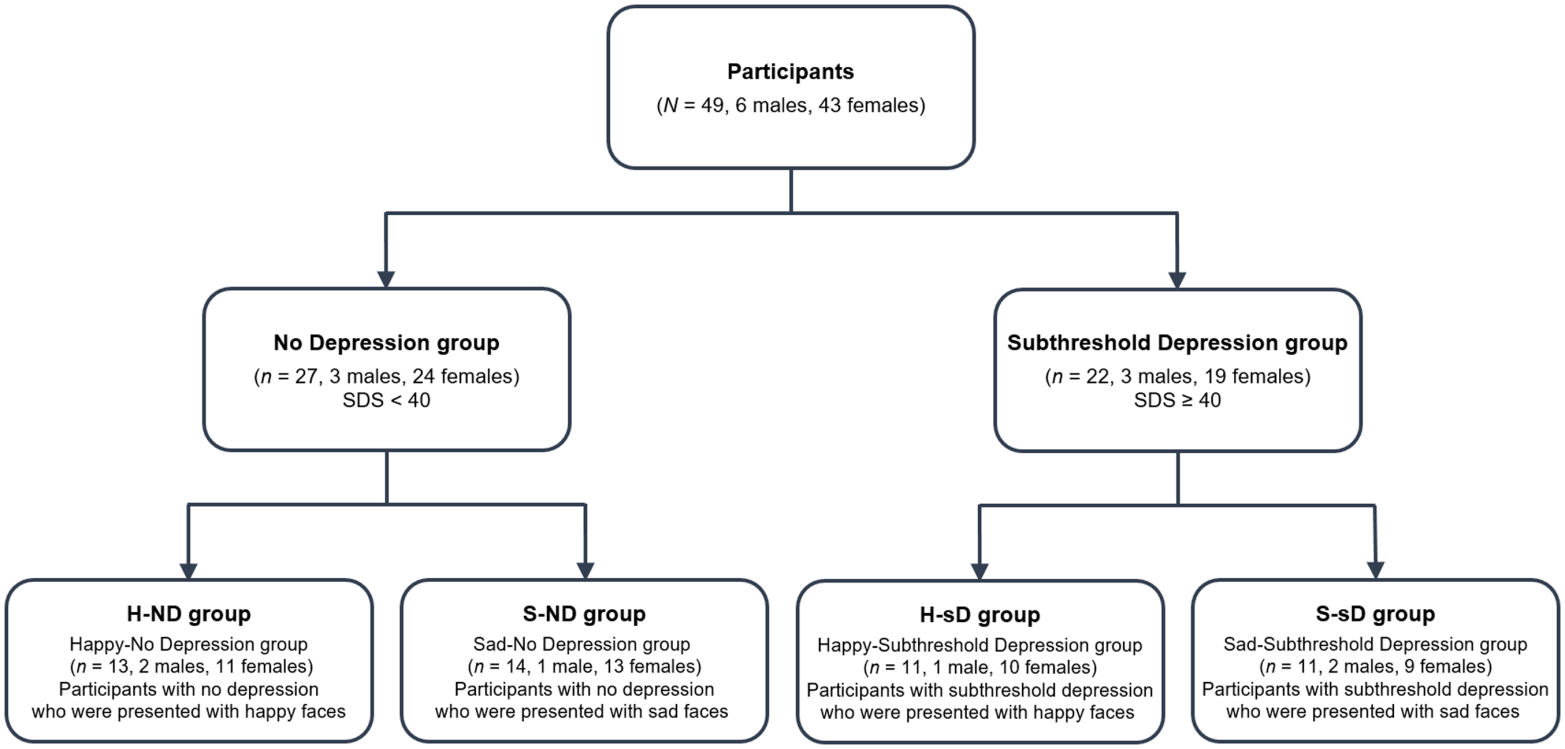
Classification of participants into groups. SDS, self-rating depression scale of Zung (1965).

They were divided into an sD group, in which participants exceeded the cutoff score (≥40) for depressive symptoms according to the Japanese version of the Self-Rating Depression Scale (SDS; Zung, 1965; Fukuda and Kobayashi, 1973), and a no depression (ND) group. Each group was further divided into those who were presented with pictures of happy faces (Happy-ND group, H-ND; Happy-sD group, H-sD) and those who were presented with sad faces (Sad-ND group, S-ND; Sad-sD group, S-sD). This resulted in a total of four groups (Figure 1). To control the confounding influence of sex and emotional empathy, we ensured that groups had the same sex ratio and a similar degree of empathy, which was measured using the Japanese version of the Questionnaire Measure of Emotional Empathy (QMEE; Mehrabian and Epstein, 1972; Kato and Takagi, 1980).

This study was approved by the Research Ethics Committee at the Graduate School of Social and Industrial Science and Technology, Tokushima University (acceptance number 162). After participants had received a description of the study, all of them signed an informed consent form. Participants received a 500 Japanese Yen (~USD 4.6) book card in remuneration for their contribution.

### Facial Stimuli

Ten faces each of happy, sad, and neutral (from six men and four women) from the ATR Facial Expression Image Database DB99 (ATR-Promotions, Inc.) were used. Facial expressions of various people were selected as facial stimuli to control for the influence of individual differences in the evaluation of facial expression (e.g., attractiveness, likability).

### Questionnaires

#### Self-Rating Depression Scale

To measure depressive tendency, the Japanese version of the SDS (Zung, 1965; Fukuda and Kobayashi, 1973) was used. The questionnaire has 20 items, such as “I feel down-hearted and blue,” and each item is rated on a scale from 1 (a little of the time) to 4 (most of the time). To make our participants evaluate their conditions correctly, item 12 was modified from “I find it easy to do the things I used to” to “I find it easy to do the things (study) I used to.”

#### Questionnaire Measure of Emotional Empathy

Dimberg and Thunberg (2012) have suggested that people who have high emotional empathy are more susceptible to emotional contagion than people who have low emotional empathy. To control for this confounding influence, we measured emotional empathy using the Japanese version of the QMEE (Mehrabian and Epstein, 1972; Kato and Takagi, 1980), and ensured that the four groups had similar levels of empathy.

This 25-item questionnaire measures emotional empathy when perceiving the emotional state of others. The questionnaire consists of three subscales, including “emotional-warmth” (10 items), “emotional-coolness” (10 items), and “emotional-susceptibility” (five items). Each item is rated on a scale from 1 (very strongly disagree) to 7 (very strongly agree). In this study, we only used the “emotional-warmth” subscale, which is often used to measure empathy (cf. Sugiyama, 2009; Hamaguchi, 2017). This subscale consists of items such as “I get very angry when I see someone being ill-treated.”

#### Mood Inventory

The Mood Inventory questionnaire was developed by Sakano et al. (1994) and it measures the five following factors: “tension and excitement,” “refreshing mood,” “fatigue,” “depressive mood,” and “anxious mood.” In this study, “refreshing mood” and “depressive mood,” which comprise items that best measure emotions related to happy and sad faces, were used as the indexes of happy and sad emotional contagion. Each factor includes eight items that are rated from 1 (strongly disagree) to 4 (strongly agree). The “refreshing mood” factor consists of items such as “I’m full of vitality,” and the “depressive mood” factor consists of items such as “I feel depressed.” Using these two factors, we asked participants to rate their mood before and after the presentation of facial stimuli to investigate the occurrence of emotional contagion of happy and sad faces. The Mood Inventory has favorable psychometric properties (Sakano et al., 1994), including adequate test-retest reliability (refreshing mood, *r*=0.62; depressive mood, *r*=0.68), internal consistency (refreshing mood, *α*=0.87; depressive mood, *α*=0.91), and concurrent validity (refreshing mood, *r*=0.83; depressive mood, *r*=0.70).

### Facial EMG

The facial EMG activity was measured using a bioelectric amplifier (AB-610J, Nihon-Kohden Corporation, Tokyo, Japan), an A/D converter (PowerLab 2/25, ADInstruments, Japan, Nagoya), and Ag/AgCl miniature surface electrodes. Following previous studies that examined facial mimicry using facial EMG (e.g., Dimberg, 1982), the activity of zygomatic major muscle was employed to assess mimicry of happy faces, and the activity of corrugator supercilii muscle was employed to assess mimicry of sad faces. Electrodes were filled with electrode paste and were bipolarly attached to the left zygomatic major and the corrugator supercilii muscle regions (Fridlund and Cacioppo, 1986). To reduce the electrode site impedance, the skin was cleaned with alcohol and rubbed with electrode paste. Facial EMG data were sampled at 1,000 samples per second with filtering cutoffs at 50 and 300Hz, and set hum filter at 60Hz. The data were rectified and integrated in 1s. We sampled integrated facial EMG data for 1s after presenting facial stimuli as the response to facial stimuli. Then, using the video data which were recording the states of participants during the presentation of facial stimuli, the artifacts (i.e., blinking, body movement, turning their eyes away, or facial movements that are clearly not considered mimicry) of recorded facial EMG data were confirmed, and the data with artifact were removed. Sampled facial EMG data without artifact were averaged in each type of facial stimuli, and we defined the difference between average of muscle activity when emotional faces were presented and average of muscle activity when neutral faces were presented as the response of facial muscle to each emotional faces.

### Procedure

We asked participants to complete the SDS and QMEE at the time of recruitment. Participants were then allocated to one of the four groups according to the results. After arriving at the laboratory, participants were informed about the purpose and procedure of the study and were asked to provide informed consent. To prevent demand characteristics/awareness effect on the results, regarding the purpose of our study, we explained abstractly to the participants, “The purpose of our study is to examine the characteristics of the physiological functions of people with depressive tendency using the experimental apparatus. In particular, we investigate how the physiological functions of people with depressive tendency change when they perceive the facial expressions of others in their daily lives.” In addition, to reduce the participants’ attention to their facial muscles during the presentation of facial stimuli, we did not tell them that they were being measured their facial muscle activities. Moreover, in accordance with Lundqvist and Dimberg (1995), to mask the purpose of our study, participants received the following explanation concerning the rating of their emotional states: “Since different participants participate at different times of the day, they may also experience different degrees of tiredness and mood. To control for differences in mood, please answer these questions.” After participants had provided written informed consent, they were asked to complete the SDS again to confirm the degree of depressive symptoms at the time of the experiment. Participants who had not been able to complete the SDS and QMEE at the time of recruitment were asked to complete both questionnaires at this point.

Participants moved to a shielded room and were attached to EMG electrodes. After that, participants sat comfortably in a chair in front of a desk. A rest period of 3min was implemented to encourage a neutral emotional state prior to experimentation. Participants were then asked to rate their current emotional state using the Mood Inventory before presentation of the stimulus slides (pre-test).

**Figure 2:**
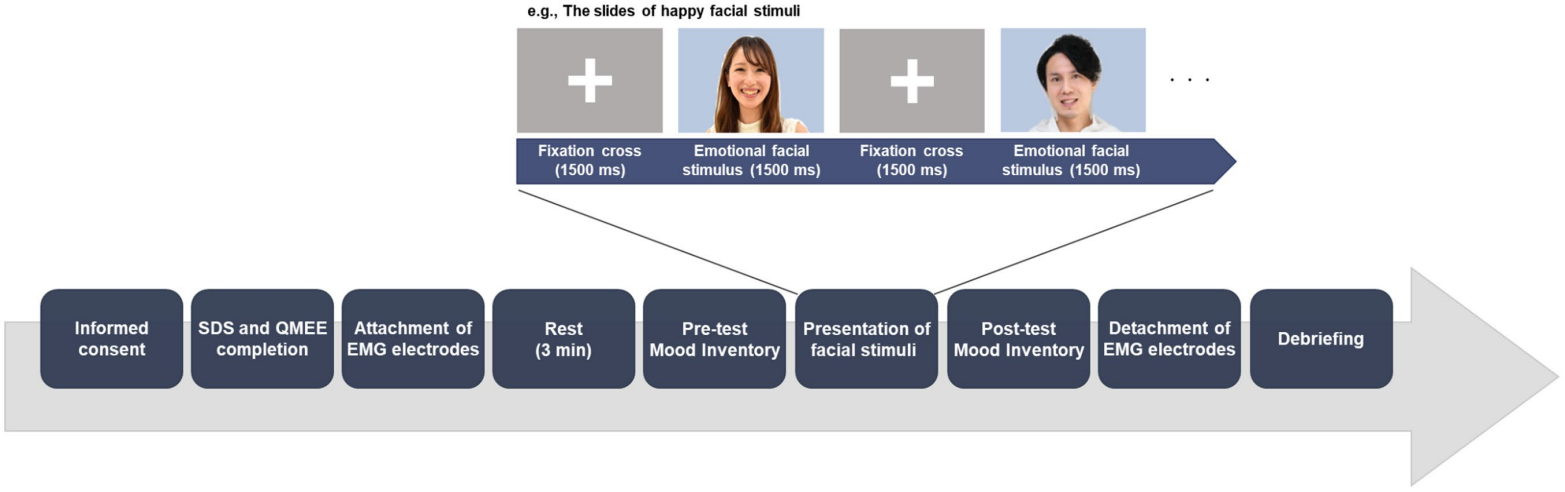
Experimental procedure. The order of presentation of emotional and neutral facial expressions was counterbalanced across participants. Immediately after the presentation of the set includes only stimuli of emotional facial expression (happy or sad), participants were asked to rate their current emotional state using the Mood Inventory (post-test). The example pictures resemble those in the experiment, but are not part of the ATR Facial Expression Image Database DB99. To maintain novelty and efficacy of the stimulus set, alternate pictures available on the public database were used with permission.

After completing the Mood Inventory, a PC was placed on the desk. The screen size was 20×35cm and participants were instructed to move the chair to a comfortable distance from the screen. Then, participants were presented with two sets of stimulus slides. One includes only neutral facial expressions the other includes only each emotional facial expression according to their group allocation (happy or sad). The order of these two sets was counterbalanced across participants. In each set, a fixation-cross was presented for 1,500ms to ensure that participants were focusing on the center of the screen, after which a facial stimulus was presented for 1,500ms. Each of the 10 faces were presented three times; that is, a total of 30 facial stimuli were presented in a randomized order per one set. In the set of facial stimuli presented earlier, one happy, one sad, and one neutral facial stimulus were presented randomly as test trials before the presentation of the stimuli of the particular facial expressions was begun. After presentation of the set includes only stimuli of emotional facial expression (happy or sad), participants were asked to rate their current emotional state using the Mood Inventory (post-test). After presenting with the two stimulus sets, EMG electrodes were detached and participants returned to the laboratory, where they received a reward and were debriefed. Figure 2 shows the procedure of our study.

### Data Analyses

We used the Statistical Package for the Social Sciences (SPSS) version 24 (IBM, Tokyo, Japan) for the statistical analyses. Two student’s *t*-tests were applied to confirm whether the SDS score was similar between the H-ND and S-ND groups, and between the H-sD and S-sD groups. A one-way ANOVA was used to assess between-group differences in the QMEE score to confirm that the empathy score was similar across the four groups. In addition, a chi-squared test was used to compare the sex ratio between the four groups.

Regarding emotional contagion, to check that the emotional states before presentation of facial stimuli were similar between the four groups, two separate one-way ANOVAs were performed for happy and sad mood scores before presentation of the facial stimuli, with the groups as between-subject factors. Since there were significantly differences between the groups, we then used the change in the two mood scores (happy and sad) from before to after stimuli presentation as the dependent variables (happy and sad mood change score) in the following analyses. Two separate two-way ANOVAs were performed for the two mood change scores, with facial stimuli (happy vs. sad) and depressive tendency (ND vs. sD) as the between-subject factors.

To examine the relationship between depressive tendency and emotional contagion, we conducted Pearson’s correlation analyses between the SDS score and the two mood change scores within each group, and between the SDS score and QMEE.

Regarding facial mimicry, two participants in the S-ND and H-sD groups were excluded from the analysis because their recording of facial muscle activity was incomplete. Therefore 47 participants were included in the analysis of facial mimicry. Two separate two-way ANOVAs were performed for the two muscle region, with facial stimuli (happy vs. sad) and depressive tendency (ND vs. sD) as the between-subject factors. For all statistical analyses, the level of significance was set at *p*<0.05.

## Results

### Demographic Characteristics

Table 1 shows descriptive statistics for each group. The *t*-tests revealed no significant differences in SDS score between the H-ND and S-ND groups [*t* (25)=0.61, *p*=0.548, *d*=0.24], or between the H-sD and S-sD groups [*t* (20)=0.26, *p*=0.798, *d*=0.11]. The one-way ANOVA revealed no differences in the QMEE score between the four groups [*F* (3, 45)=0.48, *p*=0.701, *𝜂*_𝑝_^2^=0.031]. In addition, the chi-squared test showed no significant differences in sex ratio between the four groups [*χ*^2^ (3)=0.921, *p*=0.820, *φ*=0.14].

**Table 1:** Demographic characteristics in the four groups.

	H-ND group (*n*=13)		S-ND group (*n*=14)		H-sD group (*n*=11)		S-sD group (*n*=11)	
	Male	Female	Male	Female	Male	Female	Male	Female
Sex	2	11	1	13	1	10	2	9
	***M***	***SD***	***M***	***SD***	***M***	***SD***	***M***	***SD***
Age	19.85	1.14	19.71	0.99	20.18	1.25	19.91	1.22
SDS	35.08	3.43	34.21	3.89	44.45	3.30	44.09	3.27
QMEE	50.85	4.34	50.79	4.98	53.00	4.84	51.82	6.59
Happy mood score (pre-test)	22.77	2.13	25.14	2.35	21.82	2.14	20.18	2.09
Happy mood score (post-test)	23.00	3.03	23.57	2.59	22.18	2.79	18.82	2.96
Sad mood score (pre-test)	9.85	2.73	8.71	1.44	11.45	3.33	12.18	3.16
Sad mood score (post-test)	9.62	2.40	9.79	2.42	9.45	2.54	13.36	5.57
Zygomatic major muscle activity (*μV*)	1.36	2.15	−0.35	0.55	0.96	1.00	0.02	0.24
Corrugator supercilii muscle activity (*μV*)	−0.35	1.03	0.55	1.55	−0.99	1.52	0.77	1.30

The data on facial muscle activity were obtained from 47 participants (S-ND group: *n*=13, H-sD group: *n*=10). The H-ND and H-SD groups were presented with pictures of happy faces, and the S-ND and S-SD groups were presented with pictures of sad faces. H-ND, happy-no depression group; S-ND, sad-no depression group; H-sD, happy-subthreshold depression group; and S-sD, sad-subthreshold depression group.

### Emotional Contagion of Happy Faces

A one-way ANOVA revealed there were significant differences in the happy mood score between the four groups before the presentation of facial stimuli [*F* (3, 45)=11.27, *p*<0.001, *𝜂*_𝑝_^2^=0.429; Table 1]. Therefore, we used the happy mood change score as the dependent variable in the following analysis.

**Figure 3:**
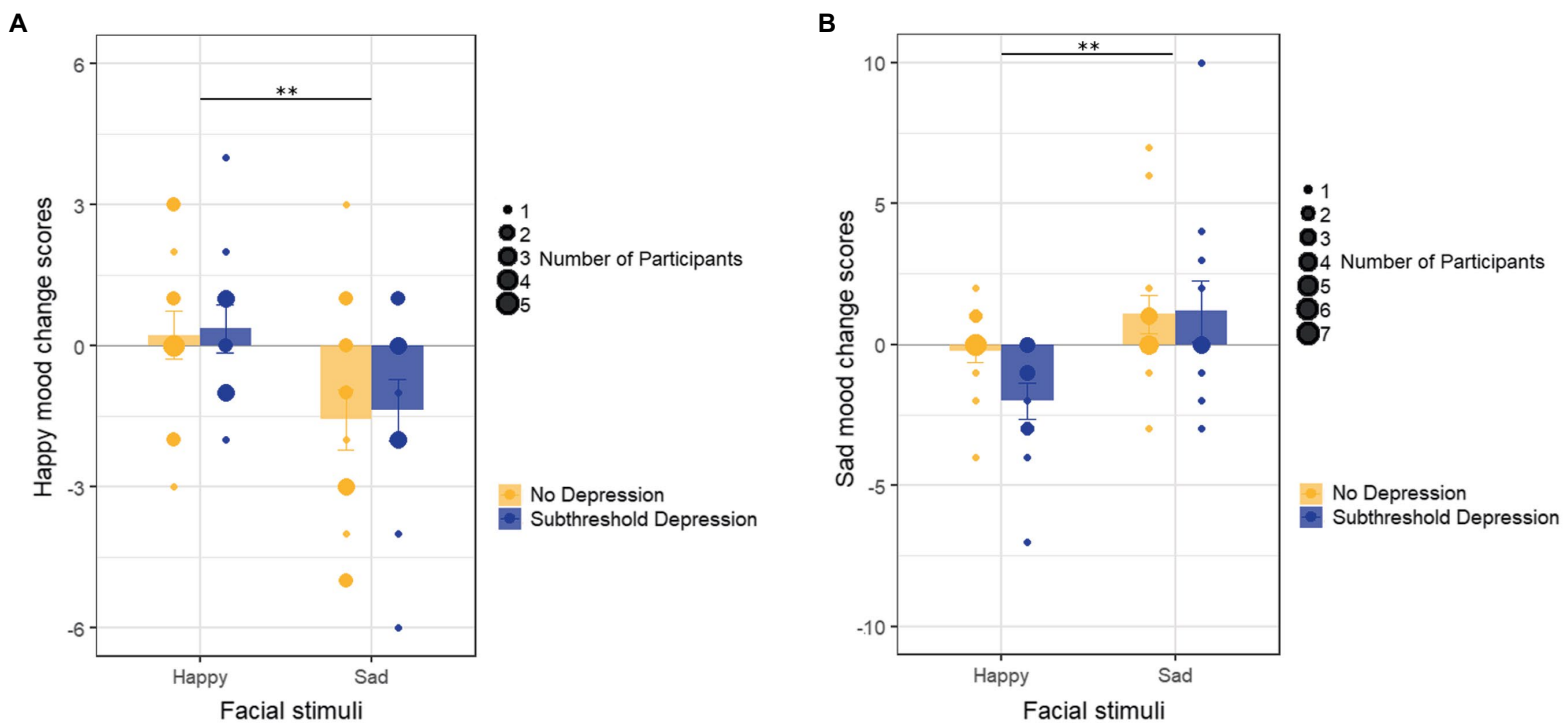
Emotional contagion in the four groups. **(A)** The changes in happy mood scores before and after presentation of facial stimuli in the four groups. **(B)** The changes in sad mood scores before and after the presentation of facial stimuli in the four groups. Error bars represent the SEs. ^**^p<0.01.

A two-way ANOVA (facial stimuli×depressive tendency) revealed a significant main effect of facial stimuli on the happy mood change score [*F* (1, 45)=8.90, *p*=0.005, *𝜂*_𝑝_^2^=0.165], whereby the happy mood change score was significantly larger in the groups presented with happy faces compared with the groups presented with sad faces (Figure 3A). There was no main effect of depressive tendency [*F* (1, 45)=0.08, *p*=0.775, *𝜂*_𝑝_^2^=0.002] and no interaction between facial stimuli and depressive tendency [*F* (1, 45)=0.004, *p*=0.950, *𝜂*_𝑝_^2^<0.001] on the happy mood change score.

### Emotional Contagion of Sad Faces

A one-way ANOVA revealed there were significant between-group differences in the sad mood score before the presentation of facial stimuli [*F* (3, 45)=4.17, *p*=0.011, *𝜂*_𝑝_^2^=0.218; Table 1]. Therefore, we used the sad mood change score as the dependent variable in the following analysis.

A two-way ANOVA (facial stimuli×depressive tendency) revealed a significant main effect of facial stimuli on the sad mood change score [*F* (1, 45)=9.55, *p*=0.003, *𝜂*_𝑝_^2^=0.175], whereby the sad mood change score was significantly larger in the groups presented with sad faces as compared with the groups presented with happy faces (Figure 3B). There was no main effect of depressive tendency [*F* (1, 45)=1.31, *p*=0.259, *𝜂*_𝑝_^2^=0.028] and no interaction between facial stimuli and depressive tendency [*F* (1, 45)=1.68, *p*=0.202, *𝜂*_𝑝_^2^=0.036] on the sad mood change score.

### Correlation Between Depressive Tendency and Emotional Contagion

There was no significant correlation between SDS score and the happy mood change score in the groups presented with happy faces (*r*=−0.10, *p*=0.649). There was no significant correlation between SDS score and the sad mood change score in the groups presented with sad faces (*r*=0.07, *p*=0.744).

In addition, there was no significant correlation between the SDS and QMEE score (*r*=0.22, *p*=0.131).

### Facial Mimicry

The activity of facial muscles in each group is shown in Table 1. Regarding mimicry of happy faces, the two-way ANOVA (facial stimuli×depressive tendency) revealed a significant main effect of facial stimuli on the activity of zygomatic major muscle [*F* (1, 43)=12.77, *p*=0.001, *𝜂*_𝑝_^2^=0.229], whereby the activity of zygomatic major muscle was significantly larger in the groups presented with happy faces compared with the groups presented with sad faces. There was no main effect of depressive tendency [*F* (1, 43)<0.01, *p*=0.951, *𝜂*_𝑝_^2^<0.001] and no interaction between facial stimuli and depressive tendency [*F* (1, 43)=1.08, *p*=0.304, *𝜂*_𝑝_^2^=0.025] on the activity of zygomatic major muscle.

Regarding mimicry of sad faces, the two-way ANOVA (facial stimuli×depressive tendency) revealed a significant main effect of facial stimuli on the activity of corrugator supercilii muscle [*F* (1, 43)=11.20, *p*=0.002, *𝜂*_𝑝_^2^=0.207], whereby the activity of corrugator supercilii muscle was significantly larger in the groups presented with sad faces compared with the groups presented with happy faces. There was no main effect of depressive tendency [*F* (1, 43)=0.29, *p*=0.596, *𝜂*_𝑝_^2^=0.007] and no interaction between facial stimuli and depressive tendency [*F* (1, 43)=1.18, *p*=0.283, *𝜂*_𝑝_^2^=0.027] on the activity of corrugator supercilii muscle.

## Discussion

The purpose of this study was to explore the usefulness of emotional contagion in modifying depressive moods in people with sD. First, we confirmed that the SDS score was not significantly different between the H-ND and S-ND groups and between the H-sD and S-sD groups, and that the QMEE score and gender ratio were not significantly different between the four groups. Thus, we can assume that these factors did not influence the emotional contagion results. Next, the groups presented with happy faces (the H-ND and H-sD groups) exhibited a slight increase in the happy mood score and a decrease in the sad mood score. The groups presented with sad faces (the S-ND and S-sD groups) exhibited an increased sad mood score and a decreased happy mood score. Moreover, there was no correlation between the SDS score and the happy mood change score of the groups presented with happy faces, or between the SDS score and the sad mood change score of the groups presented with sad faces. The SDS score was not correlated with the QMEE score. In addtion, regarding facial mimicry, the groups presented with happy faces (the H-ND and H-sD groups) mimicked happy faces, and the groups presented with sad faces (the S-ND and S-sD groups) mimicked sad faces.

Consistent with previous studies (Lundqvist and Dimberg, 1995; Hess and Blairy, 2001; Olszanowski et al., 2019), participants’ emotional states were consistent with the perceived facial expressions. Emotional contagion occurred regardless of depressive tendency. The result that both ND group and sD group mimicked the facial expressions they perceived supports that emotional contagion also occurs in people with sD because facial mimicry is considered to be associated with the occurrence of emotional contagion (e.g., Hatfield et al., 2014). Moreover, our results showed the effects of emotional contagion on mood by using a more sophisticated experimental design than previous studies. To the best of our knowledge, this is the first study to report that emotional contagion affects moods in people with sD. There are many people who are not diagnosed with depression, even though they exhibit significant depressive symptoms (Bertha and Balázs, 2013). They feel psychological distress, and thus a method to improve their depressive symptoms is needed. Our results indicate that the positive facial expressions such as happy of those around people with sD, such as family and friends, could slightly increase positive mood and relieve negative mood in people with sD. On the other hand, the negative facial expressions such as sad of those around people with sD could increase negative mood and decrease positive mood in people with sD. These findings indicate the modulating effects of emotional contagion on depressive moods in people with sD. Although the effect might be mild, emotional contagion could be used to relieve depressive moods in people with sD who have not been diagnosed with a disorder but are suffering depressive symptoms. In addtion, emotional contagion is an ability that is inherent to humans and allows us to adapt to social situations, and it does not require training or cost. Thus, individuals who come into contact with people with sD in their daily lives could use emotional contagion strategies to help relieve psychological distress and prevent the worsening of depressive symptoms in people with sD.

Furthermore, although it requires some thought, our results have implications for the attitude of healthcare professionals in clinical settings, such as psychotherapists, nurses, and doctors. There are likely to be many people with subthreshold depression in clinical setting, the facial expressions of health care practitioners may play an important role in relieving their depressive moods and facilitating treatment. Consistent with this, Padesky and Mooney (2012) mentioned that in psychotherapy focusing on the clients’ strengths, the increased use of smiling by therapists encourages clients to reveal their positive qualities. In addition, our results support the conventional view that a warm attitude on the part of counselors is important for the improvement of psychological symptoms of clients (Rogers, 1957; Lambert and Barley, 2001).

However, it is important to consider the context in order to utilize the effect of such emotional contagion. For example, when people with sD is talking about a negative event, it is not appropriate to listen to the event with a smile. In fact, Yabar and Hess (2007) showed that participants were more likely to have positive impressions of a partner who showed a sympathetic expression of sadness when they were talking about a sad event. Therefore, it should consider the appropriate timing because their positive facial expression does not always make people with sD feel better or promote the treatment.

Our study has several limitations. First, our results were obtained from people with a relatively mild depressive tendency, whose average SDS score was less than 45. This may limit the generalizability of our findings to people with moderate depressive symptoms and clinical groups with more severe depressive symptoms. Moreover, the small sample size is considered a limitation and should be targeted at a larger population. Finally, our study used static facial stimuli that were presented to participants to induce emotional contagion. However, as mentioned by Tamura and Kameda (2006), perceiving static facial expressions of others is unlikely to simulate real-world situations. Therefore, future studies should conduct research in more realistic settings by using dynamic facial stimuli to confirm the effects of emotional contagion.

## Data Availability Statement

The datasets generated for this study are available on request to the corresponding author.

## Ethics Statement

The studies involving human participants were reviewed and approved by The Research Ethics Committee at the Graduate School of Social and Industrial Science and Technology, Tokushima University. Written informed consent from the participants’ legal guardian/next of kin was not required to participate in this study in accordance with the national legislation and the institutional requirements.

## Author Contributions

YY and TY conceived and designed the experiments, wrote the manuscript, and have approved the final manuscript. YY performed the experiments and analyzed the data. All authors contributed to the article and approved the submitted version.

## Funding

This work was supported by JSPS KAKENHI (grant number 18K13323 and 21H00949).

## Conflict of Interest

The authors declare that the research was conducted in the absence of any commercial or financial relationships that could be construed as a potential conflict of interest.

## Publisher’s Note

All claims expressed in this article are solely those of the authors and do not necessarily represent those of their affiliated organizations, or those of the publisher, the editors and the reviewers. Any product that may be evaluated in this article, or claim that may be made by its manufacturer, is not guaranteed or endorsed by the publisher.

## References

[ref1] Balázs J. Miklõsi M. Keresztény Á. Hoven C. W. Carli V. Wasserman C.. (2013). Adolescent subthreshold-depression and anxiety: psychopathology, functional impairment and increased suicide risk. J. Child Psychol. Psychiatry 54, 670–677. doi: 10.1111/jcpp.12016, PMID: 23330982

[ref2] Bertha E. A. Balázs J. (2013). Subthreshold depression in adolescence: a systematic review. Eur. Child Adolesc. Psychiatry 22, 589–603. doi: 10.1007/s00787-013-0411-0, PMID: 23579389

[ref3] Cuijpers P. Smit F. (2004). Subthreshold depression as a risk indicator for major depressive disorder: a systematic review of prospective studies. Acta Psychiatr. Scand. 109, 325–331. doi: 10.1111/j.1600-0447.2004.00301.x, PMID: 15049768

[ref4] Cuijpers P. Smit F. Van Straten A. (2007). Psychological treatments of subthreshold depression: a meta-analytic review. Acta Psychiatr. Scand. 115, 434–441. doi: 10.1111/j.1600-0447.2007.00998.x, PMID: 17498154

[ref5] Demenescu L. R. Kortekaas R. den Boer J. A. Aleman A. (2010). Impaired attribution of emotion to facial expressions in anxiety and major depression. PLoS One 5:e15058. doi: 10.1371/journal.pone.0015058, PMID: 21152015 PMC2995734

[ref6] Dimberg U. (1982). Facial reactions to facial expressions. Psychophysiology 19, 643–647. doi: 10.1111/j.1469-8986.1982.tb02516.x, PMID: 7178381

[ref7] Dimberg U. Thunberg M. (2012). Empathy, emotional contagion, and rapid facial reactions to angry and happy facial expressions. Psych J. 1, 118–127. doi: 10.1002/pchj.4, PMID: 26272762

[ref8] Fridlund A. J. Cacioppo J. T. (1986). Guidelines for human electromyographic research. Psychophysiology 23, 567–589. doi: 10.1111/j.1469-8986.1986.tb00676.x, PMID: 3809364

[ref9] Fukuda K. Kobayashi S. (1973). A study on a self rating depression scale. Psychiatr. Neurol. 75, 673–679. 4798819

[ref10] Hamaguchi Y. (2017). Development of self-report proactive and reactive aggressiveness scales for university students (SPRAS-U): relationship with aggressive behavior. Jpn. J. Educ. Psychol. 65, 248–264. doi: 10.5926/jjep.65.248

[ref11] Hatfield E. Bensman L. Thornton P. D. Rapson R. L. (2014). New perspectives on emotional contagion: a review of classic and recent research on facial mimicry and contagion. Int. Int. J. Pers. Relat. 8, 159–179. doi: 10.5964/ijpr.v8i2.162

[ref12] Hess U. Blairy S. (2001). Facial mimicry and emotional contagion to dynamic emotional facial expressions and their influence on decoding accuracy. Int. J. Psychophysiol. 40, 129–141. doi: 10.1016/S0167-8760(00)00161-6, PMID: 11165351

[ref31] Kato T. Takagi H. (1980). Characteristics of the emotional empathy in adolescence. Tsukuba Psychol. Res. 2, 33–42.

[ref13] Lambert M. J. Barley D. E. (2001). Research summary on the therapeutic relationship and psychotherapy outcome. Psychotherapy 38, 357–361. doi: 10.1037/0033-3204.38.4.357

[ref14] Lundqvist L. O. Dimberg U. (1995). Facial expressions are contagious. J. Psychophysiol. 9, 203–211.

[ref15] Mehrabian A. Epstein N. (1972). A measure of emotional empathy. J. Pers. 40, 525–543. doi: 10.1111/j.1467-6494.1972.tb00078.x, PMID: 4642390

[ref16] Naganuma Y. Tachimori H. Kawakami N. Takeshima T. Ono Y. Uda H.. (2006). Twelve-month use of mental health services in four areas in Japan: findings from the world mental health Japan survey 2002-2003. Psychiatry Clin. Neurosci. 60, 240–248. doi: 10.1111/j.1440-1819.2006.01492.x, PMID: 16594950

[ref17] Olszanowski M. Wróbel M. Hess U. (2019). Mimicking and sharing emotions: a re-examination of the link between facial mimicry and emotional contagion. Cognit. Emot. 34, 367–376. doi: 10.1080/02699931.2019.1611543, PMID: 31072246

[ref18] Otsubo T. Tanaka K. Koda R. Shinoda J. Sano N. Tanaka S.. (2005). Reliability and validity of Japanese version of the mini-international neuropsychiatric interview. Psychiatry Clin. Neurosci. 59, 517–526. doi: 10.1111/j.1440-1819.2005.01408.x, PMID: 16194252

[ref19] Padesky C. A. Mooney K. A. (2012). Strengths-based cognitive-behavioural therapy: a four-step model to build resilience. Clin. Psychol. Psychother. 19, 283–290. doi: 10.1002/cpp.1795, PMID: 22653834

[ref20] Rogers C. R. (1957). The necessary and sufficient conditions of therapeutic personality change. J. Consult. Psychol. 21, 95–103. doi: 10.1037/h0045357, PMID: 13416422

[ref21] Sakano Y. Fukui T. Kumano H. Horie H. Kawahara K. Yamamoto H.. (1994). Development and validation of a new mood inventory. Jpn. J. Psychosom. Med. 34, 629–636. doi: 10.15064/jjpm.34.8_629

[ref22] Santos V. Paes F. Pereira V. Arias-Carrión O. Silva A. C. Carta M. G.. (2013). The role of positive emotion and contributions of positive psychology in depression treatment: systematic review. Clin. Pract. Epidemiol. Ment. Health 9, 221–237. doi: 10.2174/1745017901309010221, PMID: 24358052 PMC3866689

[ref23] Sheehan D. V. Lecrubier Y. Sheehan K. H. Amorim P. Janavs J. Weiller E.. (1998). The mini-international neuropsychiatric interview (M.I.N.I.): the development and validation of a structured diagnostic psychiatric interview for DSM-IV and ICD-10. J. Clin. Psychiatry 59, 22–33. PMID: 9881538

[ref24] Shiraishi S. (2005). Cognitive therapy for reducing and preventing depressive moods: a practical study with undergraduates. Jpn. J. Educ. Psychol. 53, 252–262. doi: 10.5926/jjep1953.53.2_252

[ref25] Sugiyama C. (2009). A study of family relationship, empathy and the self-esteem of student nurses. Mater. Health 49, 484–491.

[ref26] Tamura R. Kameda T. (2006). Are facial expressions contagious in the Japanese? Shinrigaku Kenkyu 77, 377–382. doi: 10.4992/jjpsy.77.377, PMID: 17447449

[ref27] Wexler B. E. Levenson L. Warrenburg S. Price L. H. (1994). Decreased perceptual sensitivity to emotion-evoking stimuli in depression. Psychiatry Res. 51, 127–138. doi: 10.1016/0165-1781(94)90032-9, PMID: 8022947

[ref28] Yabar Y. Hess U. (2007). Display of empathy and perception of out-group members. New Zeal. J. Psychol. 36, 42–49.

[ref29] Yoshikawa E. Taniguchi T. Nakamura-Taira N. Ishiguro S. Matsumura H. (2017). Factors associated with unwillingness to seek professional help for depression: a web-based survey. BMC Res. Notes 10:673. doi: 10.1186/s13104-017-3010-1, PMID: 29202791 PMC5716254

[ref30] Zung W. W. K. (1965). A self-rating depression scale. Arch. Gen. Psychiatry 12, 63–70. doi: 10.1001/archpsyc.1965.01720310065008, PMID: 14221692

